# Avian Influenza

**DOI:** 10.3201/eid1503.081661

**Published:** 2009-03

**Authors:** Hitoshi Oshitani

**Affiliations:** Tohoku University Graduate School of Medicine, Sendai, Japan

**Keywords:** Avian influenza, H5N1, pandemic, virulence, book review

Avian influenza, caused by influenza virus A (H5N1), continues to be a source of outbreaks among avian species and of sporadic human cases that result in a high case-fatality rate. These historically unprecedented outbreaks have raised serious global concerns for both animal health and human health. Significant progress in the research of avian influenza has occurred in the past decade, but unanswered questions remain. How does avian influenza cross species barriers and acquire transmissibility among humans? How can we minimize the risk of emergence of a pandemic virus? Will subtype H5N1 maintain its virulence in humans when it becomes a pandemic virus? This book helps readers understand what is known and what remains to be known about avian influenza.

The book contains 19 articles written by leaders in avian influenza research. The authors provide a comprehensive and updated review of current knowledge on avian influenza, with particular emphasis on H5N1. The articles cover various aspects of avian influenza, including its epidemiology and ecology as well as control strategies for potential outbreaks of avian influenza in Asia and Europe. Some articles describe the molecular mechanisms of interspecies transmission and virulence in birds and humans. Both interspecies transmission and virulence are determined by many molecular changes in different genes, but the mechanisms for interspecies transmission and virulence are not completely understood. Other articles address timely and important issues such as vaccine development and antiviral resistance.

All pandemic influenza viruses in humans originated from avian influenza viruses. Understanding how an avian virus can become a pandemic virus that causes devastating effects on human health is critical. This book is a valuable reference for scientists and public health specialists who work in either animal health or human health.

